# A Neural Learning Approach for a Data-Driven Nonlinear Error Correction Model

**DOI:** 10.1155/2023/5884314

**Published:** 2023-01-23

**Authors:** Xi Fang, Nan Yang

**Affiliations:** ^1^School of Statistics and Management, Shanghai University of Finance and Economics, Shanghai, China; ^2^PICC Asset Management Company Limited, Shanghai, China

## Abstract

A nonlinear error correction model (ECM) is developed to fit nonlinear relationships between the nonstationary time series in a cointegration relationship. Different from the previous parametric methods, this paper constructs a hybrid neural network to learn the nonlinear error correction model by combining a linear recurrent neural network with a multilayer BP network. The network learning algorithm is given by using the gradient descent method and error back propagation. Based on the principle of data-driven, all network parameters can be obtained through the network learning and training. The daily data of gold price and the US dollar index in 2021 were used to verify this proposed nonlinear ECM neural learning method and the results were compared by the likelihood ratio Chi-square test. Simulation results show that the proposed data-driven nonlinear error correction neural learning method can improve goodness of fit statistical significantly of complex nonlinear relationship between time series.

## 1. Introduction

The error correction model (ECM) proposed by Davidson et al. [1] is a regression model established by using the difference between variables and error correction terms. According to the Granger theorem proposed by Engle and Granger [2], if there is a cointegration relationship between nonstationary variables, an ECM can be established to reflect the relationship between long-term equilibrium and short-term fluctuations among variables. ECM combines the long-term equilibrium relation with the short-term disequilibrium fluctuation in the variable series, improves the stability of the series prediction model, and effectively avoids the pseudoregression problem. ECM not only makes full use of the possible equilibrium relationship between nonstationary variables but also retains the economic significance of variables. It has become a classical model for analyzing nonstationary time series and has been widely studied and applied. This issue has been addressed by several authors in their books such as Baltagi [3] and Enders [4].

The application of ECM in time series analysis has achieved significant results. Hall et al. [5] established an ECM for house prices in the UK. This model can switch whether to carry out disequilibrium correction through the adjustment of coefficients. Kulendran and King [6] successfully used an error correction and time series model to forecast international quarterly tourist flow and pointed out that the performance of the error correction model can be improved by improving the decision-making methods of nonstationary and seasonal modelling. Cook [7] studied the generation law of the potential prediction deviation of asymmetric ECM. By dividing the error correction term, it is revealed that the prediction deviation has regularity in the prediction space. Zhang et al. [8] analyzed the forecast price of electric futures by using the ECM. The model includes not only lag variables such as futures price and spot price but also the long-term relationship between futures price and spot price. Based on the cointegration theory, Yan and Zhao [9] constructed an ECM on GDP of China, which accurately reflects the actual situation. Zhang and Zhu [10] studied the relationship between real estate investment and regional economic development in Chengdu, China. The cointegration model describes the long-term equilibrium relationship between variables, and the error correction model reflects the short-term unbalanced relationship between variables. The results show that real estate investment and economic growth in Chengdu are subject to short-term fluctuations and long-term equilibrium. Kim et al. [11] used ECM to predict the logarithmic rate of return of bitcoin (BTC), analyzed how BTC is affected by other coins, and carried out Granger causality tests on 14 cryptocurrencies. Liang [12] used ECM to find that the relationship between bitcoin yield and relevant indicators to measure monetary function is not significant, which denies the original assumption that bitcoin can bear monetary function, indicating that bitcoin does not have the ability and potential to bear monetary function.

In order to make ECM more widely used and adapt to more time series characteristics, many literatures have carried out theoretical research on ECM. Xu et al. [13] studied a static source error correction model based on MATLAB and Simulink. Jochmann et al. [14] developed a random search variable selection method for a vector error correction model and successfully applied it to the UK macroeconomic modelling. Compared with the current popular regression and vector autoregressive models, this method can break through many possible restrictions of cointegration space. Hong et al. [15] studied the influence of measurement errors on the analysis of vector process error correction model (ECM) and proposed a method using an instrumental variable (IV) to obtain the asymptotic distribution of reduced rank estimation so as to eliminate the adverse effect of endogeneity.

However, the traditional ECM still adopts linear regression. If there is a nonlinear relationship between variables, it is difficult to be fully captured by the model, which limits the application scope of ECM to a certain extent. Although the relationship between cointegration and error correction (EC) models is well described in a linear environment, its expansion in a nonlinear environment is still a challenge.

Oliveira et al. [16] proposed a hybrid optimization error correction system for time series prediction, which uses the linear model of time series, the nonlinear model of error series, and the combination prediction of different methods. The system searches for the linear and nonlinear components and the best parameters of the combination method through a particle swarm optimization algorithm. This method has achieved good results in time series prediction. Berger [17] estimated an ECM of savings and investment. The model allows for the distinction between short-term and long-term capital flows, and its parameters are allowed to change over time, which is estimated by the Kalman filter and maximum likelihood technology. Saikkonen [18] studied the Granger representation theorem against the background of the general nonlinear vector autoregressive error correction model. The model considers nonlinear autoregressive conditional heteroscedasticity, and the conditional distribution involved can be a general type of mixed distribution. Omay et al. [19] proposed a cointegration test method based on nonlinear error correction in the panel data. Escribano and Mira [20] studied the nonlinear error model, proposed a theoretical framework based on the concept of near epoch dependence (NED), and partially extended the Granger representation theorem to the nonlinear case. Psaradakis and Spagnolo [21] studied the ECM for nonlinear and discontinuous adjustment of long-term equilibrium, proposed a nonlinear error correction model with regional switching, and analyzes its prediction performance. The research shows that if the nonlinear model is used properly, considerable benefits can be obtained, especially when the imbalance adjustment is strong and/or the parameter change range is relatively large. Ma et al. [22] proposed a nonlinear multivariate spatiotemporal threshold vector error correction model for short-term traffic state prediction by using the cointegration theory and method with error correction mechanism. Through a threshold switching mechanism, the spatial cross-correlation information is combined with the piecewise linear vector error correction model to solve the problem of unknown structural changes in traffic time series. Song and Lei [23] used the nonlinear ECM to better explain the short-term dynamic mechanism of China's broad money demand and analyzed the money demand function under the condition of an open economy by introducing the variable of the exchange rate.

However, it is not easy to model a nonlinear model by the parametric method. At present, intelligent computing methods have been successfully used to deal with nonlinear time series. Hornik et al. [24] have proved that neural networks have the ability of arbitrary approximation to nonlinear dynamics. Various neural networks and methods combined with other models are applied to nonlinear time series analysis. Based on cointegration and Granger causality analysis, Haefke and Helmenstein [25] constructed a linear and neural network error correction model of the Austrian initial public offering index (IPOX_ATX_). The neural network adopts an enhanced feed-forward structure, takes the Schwartz information criterion as the estimator for predicting risk, and uses the significant relationship between IPOX_ATX_ and the Austrian stock market index ATX to predict IPOX_ATX_. Zhu et al. [26] proposed a method combining neural networks (NNs) and data assimilation (DA). Aiming at the uncertainty of the structural model, the assimilation process and prediction results of time series can be improved. Wu et al. [27] aimed at the consumer price index (CPI) series in Beijing, and using the advantages of the ARIMA time series model in linear space prediction and the BP neural network model in nonlinear space prediction, a combined differential autoregressive moving average (ARIMA) prediction model with BP network error correction was established.

As we all know, statistics is the art and science of collecting data, analyzing data, and inferring based on data, including parameter estimation, hypothesis testing, regression analysis, factor analysis, time series, nonparametric statistics. Traditional statistics mainly develops on the basis of probability theory to establish a mathematical model, collect data from an observed system, carry out quantitative analysis, and then infer and forecast so as to provide a basis and reference for relevant decisions. Establishing accurate mathematical models is the most important task in traditional statistical methods. However, although traditional statistical methods have the great advantage of being interpretable, model-based methods often have large deviations when dealing with complex systems, and it is very difficult or even impossible to establish accurate mathematical models of observation systems. Moreover, in the era of big data, great changes have taken place in the concepts of sample, data type, data acquisition, quantitative methods, and analysis methods. Therefore, in the era of big data, statistics are required to solve much more complex problems, thus putting forward higher requirements for statistics. Under such situation, it is significantly meaningful to explore and develop data-driven-based statistical methods.

Based on the data-driven method, only the input and output data of the observation system are used for direct quantitative analysis, which can break the dependence of the traditional statistical theory on the mathematical model and overcome the complex dynamic modeling and robustness problems. Data-driven methods are widely developed in control engineering, fault diagnosis, production management, and other fields. For example, in control engineering, many different data-driven methods have been studied so far, typically PID control, iterative learning control (ILC), model-free adaptive control (MFAC), etc. Especially in the recent past, data-driven approaches combining self-attention mechanisms and generative adversarial network (GAN) have been studied in depth and have resulted in remarkable applications. Hu et al. [28] developed the self-attention-based machine theory of mind for electric vehicle charging demand forecast. A short-term probabilistic charging demand forecast model is suggested to address the problem of estimating future charging demand quantiles of a charging station 15 min ahead. Real-world-data-based case studies have demonstrated its superiority in electric vehicle charging demand forecast over state-of-the-arts. Hu et al. [29] also proposed electrochemical-theory-guided modelling of the conditional GAN to improve both point and probabilistic battery calendar ageing forecasts. By using GAN's ability to learn arbitrarily complex distributions, the Capacity Forecast GAN (CFGAN) is proposed to approximate all the possible joint distributions. By using electrochemical knowledge as the guidelines for CFGAN's crucial part design, CFGAN provides a satisfying consistency between knowledge and data, making it both knowledge-driven and data-driven.

As widely known, “black-box features” are the shortcoming of machine learning. Interpretability is an important research direction for machine learning approaches. Liu et al. [30] made extraordinary and meaningful contributions. An interpretable machine learning framework that could effectively predict battery product properties and explain dynamic effects is proposed which also provides interactions of manufacturing parameters. Because no specific knowledge of battery manufacturing mechanisms is required, this data-driven framework can be easily adopted by engineers. The work assists engineers in drawing critical insights about underlying complicated battery material and manufacturing behaviour, and further contributing to smart control of battery manufacturing. Liu et al. [31] have developed an ensemble learning approach that has superiority in accuracy, interpretability, and data-driven nature. The effects of component parameters from the mixing stage on the manufactured results of Li-ion battery electrodes are scrutinized via classification modeling. The proposed effective ensemble learning framework based on RUBoost can compensate for the category imbalance issue and classify three key quality indicators.

This paper will also establish the error correction model (ECM) between the US dollar index and gold price based on cointegration analysis and use the combined network of a linear recursive neural network and a multilayer BP neural network to fit the nonlinear relationship between the US dollar index and gold price. The main difference between this paper and the existing work is the development of data-driven methods within the framework of traditional statistical theories. The proposed method not only promotes the application of data-driven methods but also makes new innovations in the conjunctive use of traditional statistical theory and data-driven solutions to take scientificity, accuracy, and interpretability into consideration.

The motivation of the research in this paper is mainly based on the following two aspects:

1. ECM can well describe the relationship between the US dollar index and gold price, but it needs to be extended to nonlinear case.
2. As both the US dollar and gold are important components of international reserves, their price linkage mechanism affects the optimal allocation of international reserves and will also be closely watched by Yang and Fang [32]. The linkage between the US dollar and the gold price is worthy of in-depth study. Several literature studies have shown that ECM can well describe the linkage between the US dollar and gold price. Zhang [33] studied the impact of the US dollar exchange rate on the gold price and described the linkage relationship between the US dollar exchange rate and gold price through cointegration analysis and ECM. Nie and Jiang [34] also established a linear ECM between the gold price and the US dollar index and studied the long-term equilibrium relationship between them.
3. However, the time series of the US dollar index and gold price have strong nonlinear characteristics. The research results of Gilmore [35] and Joy [36] show that the gold price series with violent fluctuations has chaotic characteristics, its multifractal intensity is time-varying, and the relationship between the gold price and US dollar index time series has nonlinear characteristics. Therefore, in order to describe the nonlinear linkage more accurately between the US dollar and gold, the ECM based on linear regression needs to be further extended to nonlinear ECM.
4. Neural networks have been widely used in nonlinear time series analysis, but neural model selection needs to comply with statistical principles.
5. Although neural network learning models and even deep learning models have been successful in the nonlinear field, the internal structure of these models is usually very complex, the operation mechanism is like a black box, and the intermediate process is difficult to be understood by humans. While this kind of nonlinear model improves the accuracy of the model, the number of parameters to be estimated often surges, which increases model instability and overfitting risk. Therefore, whether the improvement of the model goodness of fit can offset the negative effects of increasing parameters is the focus of this paper.
6. This paper combines the neural network learning method with the traditional linear statistical model. Not only is the goodness of fit of the model evaluated by multiple indexes but also the nested model statistical test is used to select the model. Only when the goodness-of-fit gain of the complex model is statistically significant compared with that of the simple model is it considered that such complexity is worthwhile. This design not only provides a comparative basis for linear and nonlinear time series models but also provides strong support for the subsequent accurate analysis of relationship between time series. It is worthy of in-depth discussion and research.

In the rest of the paper, Section 2 describes the proposed data-driven nonlinear ECM with neural learning approach and its implementation method.  Section 3 presents the simulation study on the nonlinear relationship analysis of the US dollar index and gold price by applying of proposed the nonlinear error correction neural learning method and showing their performance in a real-world case study. In Section 4, we make some concluding remarks.

## 2. Methodology

In this section, we explain the neural learning methods for data-driven nonlinear ECM after introducing model-based ECM.

### 2.1. Model-Based ECM

ECM is a specific form of regression model for nonstationary cointegration time series. After verifying whether there is a cointegration relationship between time series variables, the variables representing the short-term effect and the deviation degree of the long-term equilibrium state can be constructed, and the parameters can be estimated by linear regression.

The cointegration test can be used to determine whether there is a long-term equilibrium relationship between time series variables, that is, to test whether the linear combination of time series variables is stationary. In this paper, the two-step cointegration test method is used for testing. This method is proposed by Engle and Granger [2].

Set *Y*_*t*_ and *X*_*t*_ represent the two time series to be analyzed, respectively. First, after verifying *Y*_*t*_ and *X*_*t*_ is a single integer sequence of the same order, the following equation is estimated by ordinary least squares (OLS):

(1) $$\begin{array}{c} Y_{t}=\alpha_{0}+\alpha_{1}X_{t}+\mu_{t}\text{,} \end{array}$$

where *μ*_*t*_ is the error term. After obtaining the estimated coefficient ${\hat{\alpha }}_{0}$ and ${\hat{\alpha }}_{1}$, the estimated value of *Y*_*t*_ can be calculated by ${\hat{Y}}_{t}={\hat{\alpha }}_{0}+{\hat{\alpha }}_{1}X_{t}$. And the disequilibrium coefficient *e*_*t*_ are also calculated by

(2) $$\begin{array}{c} e_{t}=Y_{t}-{\hat{Y}}_{t}\text{.} \end{array}$$

Secondly, the Dickey–Fuller (DF) test or Augmented Dickey–Fuller (ADF) test is used to test the stability of *e*_*t*_. If *Y*_*t*_ and *X*_*t*_ are integral sequences of order *d*, and *e*_*t*_ is integral sequences of order *d* − *b*, *Y*_*t*_ and *X*_*t*_ are considered as cointegration of order (*d*, *b*). In the study of the long-term equilibrium relationship between gold price and US dollar index, Nie and Jiang [34] adopted the ADF cointegration test for their index sequence and found that their logarithmic sequence was nonstationary, their first-order difference was stationary, and both were first-order integration variables. Moreover, they found that their residuals had first-order autocorrelation but no second-order autocorrelation. As a result, the following error correction model with first-order lag is presented as follows:

(3) $$\begin{array}{c} ∆Y_{t}=\beta_{0}+\gamma e_{t-1}+\beta_{1}∆X_{t}+\beta_{2}∆Y_{t-1}+\beta_{3}∆X_{t-1}+\epsilon_{t}\text{,} \end{array}$$

where *Y*_*t*_ stands for gold price, and *X*_*t*_ stands for US dollar index. ∆*Y*_*t*_=*Y*_*t*_ − *Y*_*t*−1_ is the dependent variable in the mode. *e*_*t*−1_ represents the deviation degree of the early long-term equilibrium state. ∆*X*_*t*_=*X*_*t*_ − *X*_*t*−1_, ∆*Y*_*t*−1_=*Y*_*t*−1_ − *Y*_*t*−2_ and ∆*X*_*t*−1_=*X*_*t*−1_ − *X*_*t*−2_ represent short-term effects. *ϵ*_*t*_ is the error term. After linear regression, it can be obtained that the Akaike information criterion (AIC) and Schwarz criterion (SC) values of the error correction model are the smallest, *R*^2^ is the largest, and there is no autocorrelation in the residual sequence. At this time, the model can be considered the best.

The advantage of the model in equation (3) is that the difference item eliminates the possible trend factors between variables, which weakens the multicollinearity and avoids the pseudo regression problem as much as possible. Compared with the conventional differential regression model ∆*Y*_*t*_=*f*(∆*X*_*t*_, *v*_*t*_), the long-term equilibrium state deviation degree sequence is estimated by *Y*_*t*_ and *X*_*t*_ horizontal values are added to the independent variables of this model, so that the estimated value ∆*Y*_*t*_ is corrected according to the previous disequilibrium degree.

Generally, the linear regression method is still used for modelling the model in equation (3). However, time series data often have strong nonlinear characteristics. In order to describe the interaction mechanism more accurately between time series with nonlinear characteristics, most nonlinear learning methods based on neural networks represent the model as a general nonlinear ECM as follows:

(4) $$\begin{array}{c} ∆Y_{t}=g\left(∆Y_{t-1},x_{t}\right)+\epsilon_{t}\text{,} \end{array}$$

where **x**_*t*_=[∆*X*_*t*_, ∆*X*_*t*−1_, *e*_*t*−1_]^*T*^ ∈ *R*^3^. Unfortunately, due to the complex structure of neural networks, no matter what learning algorithm is adopted, people cannot carry out statistical analysis of the network process. As in Li's [37] work, nonlinear ECM is expressed in this paper by combining equations (3) and (4) as follows:

(5) $$\begin{array}{c} ∆Y_{t}=\beta_{0}+g\left(e_{t-1}\right)+\beta_{1}∆X_{t}+\beta_{2}∆Y_{t-1}+\beta_{3}∆X_{t-1}+\epsilon_{t}\text{,} \end{array}$$

where *g*(*e*_*t*−1_) describes the adjusting effect of the last cointegration residue *e*_*t*−1_ on ∆*Y*_*t*_. Its regulatory effect depends on the functional form of *g*(∙). In the work by Li [37], taking the commonly used smooth transformation autoregression (STAR) nonlinear structure model as an example, equation (5) is expressed as follows:

(6) $$\begin{array}{c} ∆Y_{t}=\beta_{0}+\rho e_{t-1}+\varphi e_{t-1}f\left(e_{t-1},\gamma ,c\right)+\beta_{1}∆X_{t}+\beta_{2}∆Y_{t-1}+\beta_{3}∆X_{t-1}+\epsilon_{t}\text{,} \end{array}$$

where *f*(∙) is the smooth transformation function, which varies continuously with *e*_*t*−1_ from [0,1]. The smoothing parameter that determines the transfer speed of this mechanism is the coefficient *γ* (which is a positive parameter), and *c* represents a threshold value, representing the point at which the transfer occurs. The exponential transformation function of ESTR model is as follows:

(7) $$\begin{array}{c} f\left(e_{t-1},\gamma ,c\right)=1-e^{-\gamma {\left(e_{t-1}-c\right)}^{2}}\text{.} \end{array}$$

The function is nonmonotonic with respect to the transformation variable, but symmetric with respect to the transformation point. The logistic transformation function of LSTR model is as follows:

(8) $$\begin{array}{c} f\left(e_{t-1},\gamma ,c\right)={\left[1+e^{-\gamma \left(e_{t-1}-c\right)}\right]}^{-1}\text{.} \end{array}$$

The function is monotonic with respect to the transformation variable, but asymmetric with respect to the transformation point. *γ* and *c* can be estimated by nonlinear least squares. If the transformation function *f*(∙)=0 or 1, the cointegration relationship is linear, otherwise, it is nonlinear. The *F* distribution can be used to test the nonlinear hypothesis.

However, the null hypothesis to test whether equation (6) is linear is the coefficient *γ*=0. Under this original assumption, the model cannot be identified because the parameters *φ* and *c* can take any value. In the work by Granger [38], Teräsvirta [39], Song, and Lei [23], the third-order Taylor expansion was carried out, and then the auxiliary regression was tested by the nonlinear hypothesis test.

Obviously, the transformation function is used to establish the nonlinear model of EMC; however, the function should change according to different time series characteristics. Further, the parameters of the transformation function are trained by regression. Once there is data updated, regression model needs to be performed again.

Under the condition that there is a cointegration relationship between time series, the error correction model is one of the most effective statistical methods, but its regression is essentially linear regression, which cannot accurately describe the nonlinear relationship between time series, and the developed transformation function and other modification modeling methods are also difficult to adapt to the complex nonlinear linkage relationship of time series. In this paper, a data-driven neural network learning method is developed to describe the nonlinear linkage relationship between time series by using the nonlinear expression ability of neural networks. This method is not only suitable for the change of data but can also meet the expression of the nonlinear relationship. More importantly, under the framework of existing statistical methods, it can obtain the test of traditional statistics, improving the scientificity and interpretability of the approach. Therefore, the data-driven approach developed in this paper is very necessary.

This paper explores the use of the nonlinear arbitrary approximation ability of neural networks to establish a nonlinear ECM. The obtained network can accurately describe the nonlinear ECM by learning. However, different from the existing nonlinear models of time series based on neural networks, this paper does not directly choose the model with the best goodness of fit but emphasizes the comparison of the model with the criterion of the statistical test.

It is worth pointing out that a hybrid neural nonlinear ECM model integrating RNN and BP networks is established by using the nonlinear representation ability of neural networks in this paper. However, we did not choose one or even other deep neural networks to learn this nonlinear relationship. This is because, on the basis of statistical tests, the existing research results have shown that the structure of nonlinear ECM between time series with a cointegration relationship is more consistent with the proposed network structure. On the other hand, the proposed method can avoid the “black box” problem of neural networks and achieve the interpretability of statistical significance for its process.

### 2.2. Data-Driven Nonlinear ECM Neural Network

The construction of the neural network data-driven nonlinear error correction learning model is mainly divided into the following three steps. Firstly, under the condition that the time series data meet the construction conditions of ECM, the ECM variables representing the short-term effect and the deviation degree of the long-term equilibrium state are constructed. Secondly, the nonlinear error correction learning model based on a two-layer recurrent neural network (RNN) and multilayer BP network and the parameter learning algorithm of the network are established. Finally, model selection among linear models and neural networks with different parameters is decided by the nested model statistical significance test.

It is well known that a three-layer feed-forward neural network can approximate any complex nonlinear continuous function if the hidden layer contains enough neurons [24]. It only takes enough hidden elements, the approximation can have arbitrarily small precision, and the number of network layers can be increased to improve the approximation performance of the network. Therefore, this paper constructs a hybrid network of linear RNNs and multilayer BP neural networks to model the nonlinear error correction model expressed in equation (5). This paper takes the example of a four-layer BP neural network, and its hidden layers are 2 layers (more layers of BP network structure can only increase its hidden layers). The network inputs are ∆**x**_*t*_ and ${\hat{e}}_{t-1}$ and input is ∆*Y*_*t*_. A first-order lag nonlinear neural network ECM is represented as follows:

(9) $$\begin{array}{c} ∆Y_{t}=\pi_{y}{∆Y}_{t-1}+\beta_{0}+\beta_{1}∆X_{t}+\pi_{x}∆X_{t-1}+\rho_{1}e_{t-1}+\rho_{2}\text{BPNN}\left(e_{t-1}\right)\text{.} \end{array}$$

**Figure 1:**
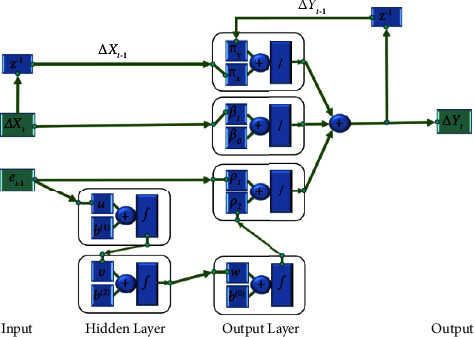
Nonlinear ECM neural network structure.

Since the output of multilayer BP neural network adopts Sigmoid function in this paper, the nonlinear part *g*(*e*_*t*−1_) of ECM is expressed in the form of linear and nonlinear combination *ρ*_1_*e*_*t*−1_+*ρ*_2_BPNN(*e*_*t*−1_). The network structure diagram of neural network input and output is shown in Figure 1.

In Figure 1, the input layer includes ∆*X*_*t*_ and its first-order lag ∆*X*_*t*−1_ and *e*_*t*−1_. The hidden layer includes two hidden layers. The *i*-th neuron input of the first hidden layer is net_*i*_, which is given as follows:

(10) $$\begin{array}{c} {\text{net}}_{i}=u_{i}e_{t-1}+b_{i}^{\left(1\right)}\text{.} \end{array}$$

After the activation function mapping, the output of the first hidden layer *h*_*i*_^(1)^ is obtained, which is given as follows:

(11) $$\begin{array}{c} h_{i}^{\left(1\right)}=f\left({net}_{i}\right)\text{.} \end{array}$$

The *j*-th input of the second hidden layer in the network is net_*j*_, which is given as follows:

(12) $$\begin{array}{c} {net}_{j}=\overset{n_{1}}{\underset{i=1}{\sum }}v_{ij}h_{i}^{\left(1\right)}+b_{j}^{\left(2\right)}\text{.} \end{array}$$

Its output is *h*_*j*_^(2)^, which is given as follows:

(13) $$\begin{array}{c} h_{j}^{\left(2\right)}=f\left({net}_{j}\right)\text{.} \end{array}$$

The output of BP neural network is as follows:

(14) $$\begin{array}{c} BPNN\left(e_{t-1}\right)=f\left({net}_{t}\right)\text{,} \end{array}$$

where

(15) $$\begin{array}{c} {net}_{t}=\overset{n_{2}}{\underset{j=1}{\sum }}w_{j}h_{j}^{\left(2\right)}+b^{\left(o\right)}\text{.} \end{array}$$

The output layer of the network includes the following three parts. The first part is the linear combination of multilayer BP output and error correction term:

(16) $$\begin{array}{c} \rho_{1}e_{t-1}+\rho_{2}BPNN\left(e_{t-1}\right)=\rho_{1}e_{t-1}+\rho_{2}f\left({net}_{t}\right)\text{.} \end{array}$$

The second part is the output of ∆*X*_*t*_ through the linear network:

(17) $$\begin{array}{c} \beta_{0}+\beta_{1}∆X_{t}\text{,} \end{array}$$

where *β*_1_ is the network weight parameter, *β*_0_ is the offset parameter of the network. The third part is the linear combination of first-order lag ∆*X*_*t*−1_ of network input and first-order lag of ∆*Y*_*t*−1_ of output as follows:

(18) $$\begin{array}{c} \pi_{y}{∆Y}_{t-1}+\pi_{x}∆X_{t-1}\text{,} \end{array}$$

where *π*_*y*_ and *π*_*x*_ are the network weight parameters.

Because the network output contains the lag term of the network output, which is equivalent to the feedback of the network, this network is a hybrid network of multilayer BP network and a linear RNN. The neural network activation function *f*(∙) selected in this paper is the Sigmoid function:

(19) $$\begin{array}{c} f\left(x\right)=\frac{1}{1+e^{-x}}\text{.} \end{array}$$

Similarly, we can use more hidden layer neural networks to improve nonlinear approximation ability.

The constructed hybrid network expressed as equation (9) is structurally similar to the ECM expressed as equation (6) with a smooth transformation autoregressive (STAR) nonlinear structure. The multilayer BP neural network is used to replace the smooth transformation function. In addition to the improvement of the nonlinear approximation expression ability of the neural network, this model can optimize the network parameters through a training and learning algorithm rather than getting all the parameters through regression. This network learning method can not only optimize the approximation coefficient but also adaptive optimization of the approximation basis function set, so it has a strong nonlinear approximation ability. Moreover, the training algorithm has better adaptability to get the approximate parameters. When the data changes, the parameters can be obtained through distributed parallel computing, and the real-time performance of the algorithm is also better. When the number of hidden layers of a neural network increases, the number of neurons in the hidden layer also increases, and the ability to model the nonlinear relationship of time series also increases. Because the nonlinear ECM is expressed by a transformation function in equation (6), the model parameters are obtained by the regression method and rely heavily on regression data. When the data changes, all the data need to participate in the regression again, and the adaptability of parameters is not as strong as the approximation coefficient obtained by neural network learning. Moreover, due to the limitation of the transformation function, its nonlinear expression ability is also restricted. At the same time, different from the neural network model in general literature, parameter set selection of network model is not only based on goodness of fit but also uses a statistical significance test to evaluate the gain of adding more parameters.

### 2.3. Neural Learning Algorithm

In this subsection, we will use the gradient descent method and error back propagation to give the network learning and training algorithm for this structure. Suppose that the output of the hybrid neural network is $∆{\tilde{Y}}_{t}$ and the loss function is the square difference between the network output value and the actual value of the dependent variable. That is

(20) $$\begin{array}{c} E_{t}=\frac{1}{2}{\left(∆{\tilde{Y}}_{t}-∆Y_{t}\right)}^{2}\text{,} \end{array}$$

(21) $$\begin{array}{c} E=\overset{T}{\underset{t=2}{\sum }}E_{t}\text{.} \end{array}$$

The error backpropagation gradient descent method is used to derive the network parameter updating formula at time *t*.

(22) $$\begin{array}{c} \frac{\partial E_{t}}{\partial \beta_{1}}=\left(∆{\tilde{Y}}_{t}-∆Y_{t}\right)\frac{\partial ∆{\tilde{Y}}_{t}}{\partial \beta_{1}}\\ =\left(∆{\tilde{Y}}_{t}-∆Y_{t}\right)\left[∆X_{t}+\pi_{y}\frac{\partial ∆{\tilde{Y}}_{t-1}}{\partial \beta_{1}}\right]\text{.} \end{array}$$

We define the error signal as follows:

(23) $$\begin{array}{c} \delta_{t}^{\beta_{1}}=\frac{\partial ∆{\tilde{Y}}_{t}}{\partial \beta_{1}}\text{,} \end{array}$$

thus

(24) $$\begin{array}{c} \frac{\partial E_{t}}{\partial \beta_{1}}=\left(∆{\tilde{Y}}_{t}-∆Y_{t}\right)\delta_{t}^{\beta_{1}}\text{,} \end{array}$$

where

(25) $$\begin{array}{c} \delta_{t}^{\beta_{1}}=∆X_{t}+\pi_{y}\delta_{t-1}^{\beta_{1}}\text{.} \end{array}$$

The error signal of time *t* implies the error at time *t* − 1, which reflects the memory ability of RNN.

Similarly, we can deduce the updated formula of other parameters of linear RNN as follows:

(26) $$\begin{array}{c} \frac{\partial E_{t}}{\partial \beta_{0}}=\left(∆{\tilde{Y}}_{t}-∆Y_{t}\right)\delta_{t}^{\beta_{0}}=\left(∆{\tilde{Y}}_{t}-∆Y_{t}\right)\left[1+\pi_{y}\delta_{t-1}^{\beta_{0}}\right]\text{,} \end{array}$$

(27) $$\begin{array}{c} \frac{\partial E_{t}}{\partial \pi_{x}}=\left(∆{\tilde{Y}}_{t}-∆Y_{t}\right)\delta_{t}^{\pi_{x}}=\left(∆{\tilde{Y}}_{t}-∆Y_{t}\right)\left[∆X_{t-1}+\pi_{y}\delta_{t-1}^{\pi_{x}}\right]\text{,} \end{array}$$

(28) $$\begin{array}{c} \frac{\partial E_{t}}{\partial \pi_{y}}=\left(∆{\tilde{Y}}_{t}-∆Y_{t}\right)\delta_{t}^{\pi_{y}}=\left(∆{\tilde{Y}}_{t}-∆Y_{t}\right)\left[∆Y_{t-1}+\pi_{y}\delta_{t-1}^{\pi_{y}}\right]\text{,} \end{array}$$

(29) $$\begin{array}{c} \frac{\partial E_{t}}{\partial \rho_{1}}=\left(∆{\tilde{Y}}_{t}-∆Y_{t}\right)\delta_{t}^{\rho_{1}}=\left(∆{\tilde{Y}}_{t}-∆Y_{t}\right)\left[{\hat{e}}_{t-1}+\pi_{y}\delta_{t-1}^{\rho_{1}}\right]\text{,} \end{array}$$

(30) $$\begin{array}{c} \frac{\partial E_{t}}{\partial \rho_{2}}=\left(∆{\tilde{Y}}_{t}-∆Y_{t}\right)\delta_{t}^{\rho_{2}}=\left(∆{\tilde{Y}}_{t}-∆Y_{t}\right)\left[\text{BPNN}\left(e_{t-1}\right)+\pi_{y}\delta_{t-1}^{\rho_{2}}\right]\text{,} \end{array}$$

for the multilayer BP network, the weight updating formula is also derived by using the error backpropagation gradient descent method.

For *w*_*j*_:

(31) $$\begin{array}{c} \frac{\partial E_{t}}{\partial w_{j}}=\left(∆{\tilde{Y}}_{t}-∆Y_{t}\right)\frac{\partial {\tilde{Y}}_{t-1}}{\partial w_{j}}\\ =\left(∆{\tilde{Y}}_{t}-∆Y_{t}\right)\left[\rho_{2}\frac{\partial BPNN\left(e_{t-1}\right)}{\partial w_{j}}\right]\\ =\left(∆{\tilde{Y}}_{t}-∆Y_{t}\right)\left[\rho_{2}f^{\prime }\left({net}_{t}\right)\frac{\partial {net}_{t}}{\partial w_{j}}\right]\text{.} \end{array}$$

We can calculate

(32) $$\begin{array}{c} \frac{\partial {net}_{t}}{\partial w_{j}}=h_{j}^{\left(2\right)}\text{.} \end{array}$$

For *b*^(*o*)^, similarly, we can obtain:

(33) $$\begin{array}{c} \frac{\partial E_{t}}{\partial b^{\left(o\right)}}=\left(∆{\tilde{Y}}_{t}-∆Y_{t}\right)\rho_{2}f^{\prime }\left({net}_{t}\right)\text{.} \end{array}$$

For *v*_*ij*_:

(34) $$\begin{array}{c} \frac{\partial E_{t}}{\partial v_{ij}}=\left(∆{\tilde{Y}}_{t}-∆Y_{t}\right)\left[\rho_{2}f^{\prime }\left({net}_{t}\right)\frac{\partial {net}_{t}}{\partial v_{ij}}\right]\text{,} \end{array}$$

(35) $$\begin{array}{c} \frac{\partial {net}_{t}}{\partial v_{ij}}=\overset{n_{2}}{\underset{j=1}{\sum }}w_{j}\frac{\partial h_{j}^{\left(2\right)}}{\partial v_{ij}}\\ =\overset{n_{2}}{\underset{j=1}{\sum }}w_{j}f^{\prime }\left({net}_{j}\right)h_{i}^{\left(1\right)}\text{.} \end{array}$$

For *b*_*j*_^(2)^, the same can be:

(36) $$\begin{array}{c} \frac{\partial E_{t}}{\partial b_{j}^{\left(2\right)}}=\left(∆{\tilde{Y}}_{t}-∆Y_{t}\right)\rho_{2}f^{\prime }\left({net}_{t}\right)\frac{\partial {net}_{t}}{\partial b_{j}^{\left(2\right)}}\text{,} \end{array}$$

(37) $$\begin{array}{c} \frac{\partial {net}_{t}}{\partial b_{j}^{\left(2\right)}}=\overset{n_{2}}{\underset{j=1}{\sum }}w_{j}f^{\prime }\left({net}_{j}\right)\text{.} \end{array}$$

For *u*_*i*_,

(38) $$\begin{array}{c} \frac{\partial E_{t}}{\partial u_{i}}=\left(∆{\tilde{Y}}_{t}-∆Y_{t}\right)\left[\rho_{2}f^{\prime }\left({net}_{t}\right)\frac{\partial {net}_{t}}{\partial u_{i}}\right]\text{,} \end{array}$$

(39) $$\begin{array}{c} \frac{\partial {net}_{t}}{\partial u_{i}}=\overset{n_{2}}{\underset{j=1}{\sum }}w_{j}f^{\prime }\left({net}_{j}\right)\frac{\partial {net}_{j}}{\partial u_{i}} \end{array}$$

(40) $$\begin{array}{c} \frac{\partial h_{i}^{\left(1\right)}}{\partial u_{i}}=f^{\prime }\left({\text{net}}_{i}\right)e_{t-1}\text{.} \end{array}$$

For *b*_*i*_^(1)^, we can get:

(41) $$\begin{array}{c} \frac{\partial E_{t}}{\partial b_{i}^{\left(1\right)}}=\left(∆{\tilde{Y}}_{t}-∆Y_{t}\right)\left[\rho_{2}f^{\prime }\left({net}_{t}\right)\frac{\partial {net}_{t}}{\partial b_{i}^{\left(1\right)}}\right]\text{,} \end{array}$$

(42) $$\begin{array}{c} \frac{\partial {net}_{t}}{\partial b_{i}^{\left(1\right)}}=\overset{n_{2}}{\underset{j=1}{\sum }}w_{j}f^{\prime }\left({net}_{j}\right)\left(\overset{n_{1}}{\underset{i=1}{\sum }}v_{ij}f^{\prime }\left({net}_{i}\right)\right)\text{.} \end{array}$$

So far, we give the updating formula of all parameters of the linear RNN and multilayer BP hybrid network.

### 2.4. Implementation of Neural Learning Methods for Nonlinear ECM

In order to avoid the influence of variable size difference on modelling, all variables in the model (∆*Y*_*t*_, ∆*X*_*t*_, and *e*_*t*_ sequence, etc.) for standardization. That is

(43) $$\begin{array}{c} X_{t}^{\prime }=\frac{X_{t}-\min\left(X\right)}{\max\left(X\right)-\min\left(X\right)}\text{.} \end{array}$$

For convenience, the processed data variable mark remains unchanged. The loss function is selected as the square difference between the network output value and the actual value of the dependent variable as equations (20) and (21). The initial value of each error signal is set to 0. Besides loss function, three commonly performance indicators, including mean square error (MSE), *R*^2^ and log *L* are presented in the simulation data to describe the network performance.

(44) $$\begin{array}{c} MSE=\frac{1}{T-1}\overset{T}{\underset{t=2}{\sum }}{\left(∆{\tilde{Y}}_{t}-∆Y_{t}\right)}^{2}\\ R^{2}={\left[cor\left(∆{\tilde{Y}}_{t},∆Y_{t}\right)\right]}^{2}\\ \log\;L=\log\;\left(f\left(∆Y_{2},∆Y_{3},\ldots ,∆Y_{t},\ldots ,∆{\left.Y_{T}\right|}_{\hat{\theta }}\right)\right)\text{,} \end{array}$$

where *f*(∆*Y*_2_, ∆*Y*_3_,…, ∆*Y*_*t*_,…, ∆*Y*_*T*_) is the joint density function of ∆*Y*_2_, ∆*Y*_3_,…, ∆*Y*_*t*_,…, ∆*Y*_*T*_ under given parameter set $\hat{\theta }$.

The algorithm solves the problem as shown as Algorithm 1.

### 2.5. Statistical Tests

In order to use the statistical test for nonlinear ECM neural network parameter set selection, the following likelihood ratio test statistics are constructed. According to the likelihood ratio test method [40, 41], under regular conditions, the likelihood ratio test statistic for testing *H*_0_ : *θ* ∈ Θ_0_ versus *H*_1_ : *θ* ∈ Θ_*c*_

(45) $$\begin{array}{c} D=2\;\log\;L\left(\hat{\theta }\right)-2\;\log\;L\left(\hat{\theta_{0}}\right)\overset{D}{⟶}\chi_{df}^{2}\text{,} \end{array}$$

where the degree of freedom df of Chi-square distribution is the difference between the number of free parameters without and with constraints. $\hat{\theta_{0}}={argmax}_{\theta \in \Theta_{0}}L\left(\theta \right)$ and $\hat{\theta }={argmax}_{\theta \in \Theta_{0}\cup \Theta_{c}}L\left(\theta \right)$. *L*(*θ*) is the likelihood function under parameter *θ*. If the *P* value calculated under the above distribution is larger than 0.05, it is considered that there is no significant difference between the two models, that is, simple model under *θ* ∈ Θ_0_ should be chosen.

In nonlinear ECM neural network, we can assume that ∆*Y*_*t*_ is normal distribution in this paper as equation (4) and *ϵ*_*t*_ ~ *N*(0, *σ*^2^). Therefor, $∆Y_{t}∼N\left(g\left(∆Y_{t-1},x_{t}\right),\sigma^{2}\right),\hat{∆Y_{t}}=g\left(∆Y_{t-1},x_{t}\right)$ can also be considered the mean of ∆*Y*_*t*_. Under the uniform variance assumption, the maximum likelihood estimation method is to find the parameters to maximize the joint distribution of ∆*Y*_*t*_, that is, to maximize the following formula:

(46) $$\begin{array}{c} f\left(∆Y_{2},∆Y_{3},\ldots ,∆Y_{t},\ldots ,∆Y_{T}\right)=\frac{e^{-\sum_{2}^{T}{\left(∆Y_{t}-\hat{∆Y_{t}}\right)}^{2}/2\sigma^{2}}}{{\left(2\pi \right)}^{T/2}\sigma^{T}}\text{,} \end{array}$$

where *T* is the number of observations.

It is easy to see that the above parameters can be obtained in two steps. For a given *σ*, in order to maximize the above formula, it is equivalent to making $\sum_{2}^{T}{\left(∆Y_{t}-\hat{∆Y_{t}}\right)}^{2}$ minimum, which is consistent with loss function of nonlinear ECM neural network. For a given $\hat{∆Y_{t}}$, we can obtain the following equation:

(47) $$\begin{array}{c} \sigma^{2}=\frac{\sum_{2}^{T}{\left(∆Y_{t}-\hat{∆Y_{t}}\right)}^{2}}{T-1}\text{.} \end{array}$$

After the above two parameters are known, the likelihood ratio test of nested model can be carried out.

(48) $$\begin{array}{c} D=2\;\log\;f\left(∆Y_{2},∆Y_{3},\ldots ,∆Y_{t},\ldots ,∆{\left.Y_{T}\right|}_{\hat{\theta }}\right)-2\;\log\;f\left(∆Y_{2},∆Y_{3},\ldots ,∆Y_{t},\ldots ,∆{\left.Y_{T}\right|}_{\hat{\theta_{0}}}\right)\overset{D}{⟶}\chi_{df.}^{2} \end{array}$$

## 3. Empirical Studies

Gold price and US dollar index have always been common varieties in international reserves and investment portfolios, while gold has the dual properties of currency and commodity, so the relationship between US dollar index and gold price has attracted academic attention. The gold price studied in this paper is London gold denominated in US dollars, which is a relatively active spot gold market in the world. The US dollar index is selected to consider the comprehensive trend of the US dollar exchange rate. The nonlinear model between the US dollar index and gold price is described by the proposed nonlinear ECM neural network. We first examine whether gold price and US dollar index conform to the cointegration relationship, and then compare and analyze the linear ECM model and nonlinear ECM neural network, respectively, to show the evolution process of the dollar-gold nonlinear relation model.

**Figure 2:**
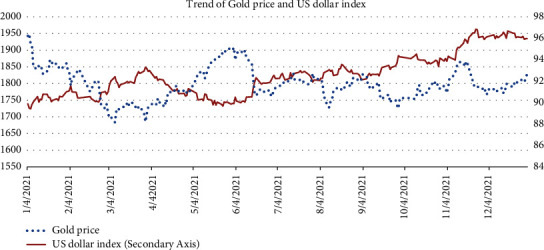
Trend of the gold price and US dollar index.

Figure 2 shows the daily frequency data of the gold price and the US dollar index from January 4, 2021, to December 31, 2021. As we can see, the relationship between these two series was constantly changing. The gold price and the US dollar index was in negative relationship in January to March as gold prices showed a sharp downward trend, while the US dollar index was just the opposite. From April to May, an inverse relation went on between the gold price and US dollar index as gold price rose and rebound while US dollar went downward. In the second half of 2021, the US dollar index maintained a gentle upward trend, and the gold price remained volatile.

### 3.1. Cointegration Test of Data

The ADF test is used to test the integration of time series in order to verify the cointegration of the dollar index and gold price. The original hypothesis of this test is that the series has a unit root, that is, it is nonstationary. When the *P* value corresponding to the DF statistic value is greater than or equal to the selected threshold, reject the original hypothesis, and it can be considered that the sequence is nonstationary; otherwise, it is stationary. The threshold value selected in this paper is 0.05.

As can be seen from Table 1, both the US dollar index series and gold price series are not stable in the selected time period, but they are stable after the first-order difference. It shows that the dollar index series and gold price meet the prerequisite of first-order cointegration and need to be further tested.

Based on the dollar index and the first-order single integration of gold price, the following long-term equilibrium model is established as follows:

(49) $$\begin{array}{c} GOLD_{t}=\alpha_{0}+\alpha_{1}USDX_{t}+\mu_{t}\text{.} \end{array}$$

The overall significance of the regression model is significant that *F* statistic is 28.64 with *P* value < 0.01 and *R*^2^ is 10.70%. The coefficients of the constant term and the US dollar index are also statistically significant, as shown in Table 2.

In the ADF stationary test of residual error in the long-term equilibrium model, the DF statistic value is −3.2177 with a *P* value <0.01. On this basis, residual stability is verified by ADF. To sum up the above analysis results, the US dollar index and gold price in 2021 meet the (1, 1) order cointegration and have the premise of building ECM model. The residual of the long-term equilibrium model will become the long-term equilibrium state deviation variable in the later model.

### 3.2. Neural Learning Results and Comparison

The simulation includes the following parameter sets: The nonlinear part of the neural network adopts the four-layer BP network, in which the number of nodes of the first and second hidden layers (*i* and *j* in subsection 2.2) is 1, 3, and 5, respectively. The ECM linear model results are also given in this paper for comparison with the nonlinear ECM neural network. The number of training epochs for the hybrid network is set to be 2000. Initialized learning step *μ* is 0.001. In order to make the nonlinear ECM neural network converge as soon as possible, the learning step is changed to 0.0001 after 1000 epochs of training.

**Figure 3:**
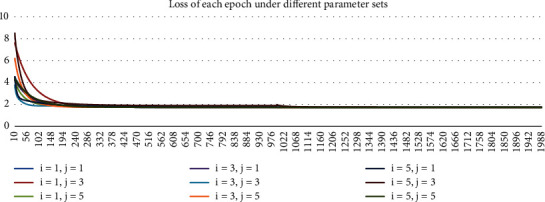
Chart of loss for each epoch under different parameter sets in 2021.

**Figure 4:**
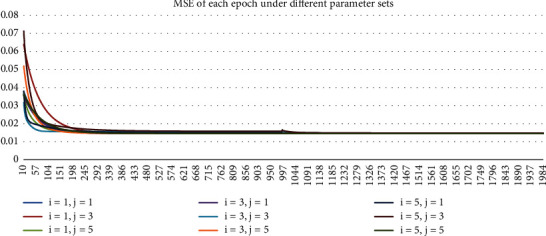
Chart of the MSE of each epoch under different parameter sets in 2021.

**Figure 5:**
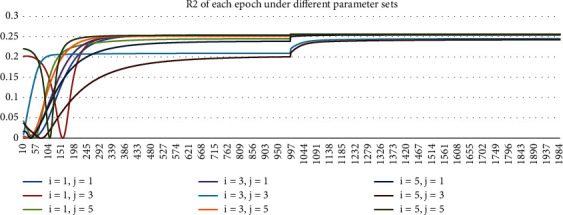
Chart of *R*^2^ of each epoch under different parameter sets in 2021.

**Figure 6:**
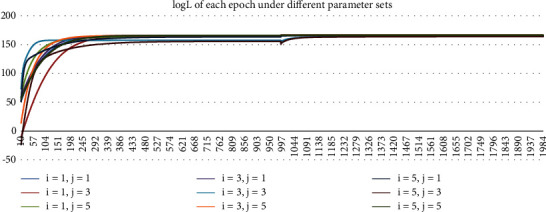
Chart of log *L* of each epoch under different parameter sets in 2021.

The training process in 2021 under different parameter sets are shown in Figures 3–6. In Figures 3 and 4, Loss and MSE no longer reduce when approaching 2000^th^ epoch. In Figures 5 and 6, *R*^2^ and log *L* no longer increase when approaching 2000^th^ epoch. As a result, the training is sufficiently complete and the network has converged. In Figures 3–6, we started from the 10^th^ epoch because the scale of the statistical indicators of the first 10 epochs changed greatly, which was caused by the random initialization parameters of the network and had nothing to do with the conclusions obtained.

The neural learning results are shown in Table 3. For the data in 2021, nonlinear ECM neural network with 1 and 5 nodes in the first and second hidden layers, respectively (model No. 4) was the best in perspective of goodness of fit. After comparing it to the most simplified nonlinear ECM neural network, which is 1 node in the first and second hidden layers, respectively, (model No. 2), the *P* value of chi-square is larger than 0.05, which means they do not have a statistically significant difference in goodness of fit. A nonlinear ECM neural network with 3 and 1 nodes in the first and second hidden layers, respectively (model No. 5), 3 and 5 nodes in first and second hidden layers, respectively (model No. 7), 5 and 5 nodes in the first and second hidden layers, respectively (model No. 10), share the same conclusion that the goodness of fit statistical indicators are better than or equal to those of model No. 2, but the difference is not statistically significant. Next, we compared model No. 2 with Linear ECM model (model No. 1). Chi-square test *P* value is 0.02, which is smaller than 0.05, and indicated model No. 2 did have statistically significant improvement in term of goodness of fit comparing with Linear ECM model.

As we can see, the goodness of fit of the ECM model with the addition of the nonlinear multilayer BP neural network in most parameter sets is better than that of the linear model in 2021. Adding the number of nodes in the first and second hidden layers does not substantially increase the goodness of fit. As shown in Table 3, log *L* only changed from 166.33 (1 node in the first and second hidden layers, respectively (model No. 2), the most simplified nonlinear multilayer BP neural network), to 166.38 (1 and 5 nodes in the first and second hidden layers, respectively (model No. 4), the nonlinear multilayer BP neural network with the best goodness of fit), while the number of parameters doubled from 12 to 24. In order to compare the models more scientifically, the likelihood ratio chi-square test was introduced to compare the complex model and the simple model. Results indicated that model No. 2 shown no statistically significant difference in goodness of fit with other nonlinear multilayer BP neural networks in more nodes. In addition, model No. 2 did statistically significantly defeated the linear model, which made it the most suitable model for the gold price and US dollar index in 2021.

### 3.3. Robustness Analysis

In order to evaluate the robustness of the proposed nonlinear ECM neural learning method and analyze its performance under different data cases, the empirical studied is carried out on generalized time period and generalized variables. In detail, besides analyzing 2021 gold price as *Y*_*t*_ and US dollar index as *X*_*t*_ in equation (3), as described in 3.2, after the cointegration test, the following simulations are also performed:

1. Generalized time period: both 2021 and 2020 are studied.
2. Generalized variables: using the US dollar index for *Y*_*t*_ and the gold price as *X*_*t*_ in equation (3).

The results are listed as in Table 4. In all 4 cases, the nonlinear ECM neural network models performs better than the linear ECM model in perspective of goodness of fit only. When likelihood ratio chi-square test was introduced to compare complex model and simple model, in most cases (3 out of 4), the advantage of nonlinear ECM neural network models is proved to be statistically significant. The necessity of the proposed nonlinear ECM neural learning method is proven. While in most cases (3 out of 4), the model with the best goodness of fit was not the comprehensively best model when both goodness of fit and model complexity were taken into consideration. The necessity of the proposed likelihood ratio chi-square test is proven.

In conclusion, the proposed nonlinear ECM neural network models can be carried out for different data cases and generalized well. The robustness and necessity of the method are verified.

## 4. Conclusions

The data-driven method combining traditional statistics under a linear paradigm with nonlinear computational intelligence is undoubtedly a potential method for financial time series data processing in the future. Aiming at the nonlinear relationship faced by the nonlinear ECM in analyzing the nonlinear relationship between gold price and US dollar index, this paper constructs a data-driven nonlinear ECM through the combination of a linear RNN and multilayer BP network and gives the corresponding neural learning algorithm to realize the accurate modelling of the nonlinear time series with a long-term equilibrium relationship. It provides a new research method for the analysis of time series by a nonlinear error correction learning model.

Different from other methods that simply use neural networks to describe nonlinear time series, the nonlinear ECM proposed in this paper is a neural learning network constructed based on the principle of statistical detection, and all network parameters will be obtained through learning. Based on empirical analysis with the gold price and US dollar index in 2021, a nonlinear ECM neural network with 1 and 5 nodes in the first and second hidden layers, respectively (model No. 4), was the best from the perspective of goodness of fit. After using the likelihood ratio chi-square test for the nested model, which means taking both goodness of fit and model complexity into consideration, a nonlinear ECM neural network with 1 node in the first and second hidden layers, respectively, is considered as comprehensively best model. The above conclusions are intuitive. In some complex economic and financial cases, the nonlinearity of time series increases, and traditional linear models need to be improved. Therefore, it is necessary to fully compare the nonlinear ECM neural network in different parameter sets with the linear model in the modelling process and then make the final model selection. However, it is not always the case that the most complex model performs best. We need to strike a balance between goodness of fit and complexity. Statistical hypothesis testing is a good method for quantitative comparison, as suggested in this paper.

The nonlinear ECM neural learning method proposed in this paper can meet the needs of traditional statistical detection so as to obtain interpretability. It not only improves the goodness of fit of time series within a certain range but also expands the application range of ECM. By combining with traditional statistical methods, a deep learning neural network learning method will be more widely used in the field of time series modelling. This method can be further extended to a real-time learning network. When new data are obtained, they participate in learning based on the original network and obtain network parameters online without all data participating in regression. It can also be easily extended to multivariable, nonlinear time series analysis methods.

The hybrid neural learning method of RNN and BP networks proposed in this paper cannot be applied to all nonlinear time series because the established learning model needs to meet the modeling conditions of ECM, namely, cointegration. Only time series that pass the cointegration test can be applied to the proposed approach. In addition, the existing deep machine learning methods, such as the combination of self-attention mechanisms and the data-driven method of the generic adversarial network, the ensemble machine learning method [28–31], will be used as references in the future research to closely combine the application conditions of ECM and develop more effective data-driven nonlinear ECM.

## Figures and Tables

**Algorithm 1:**
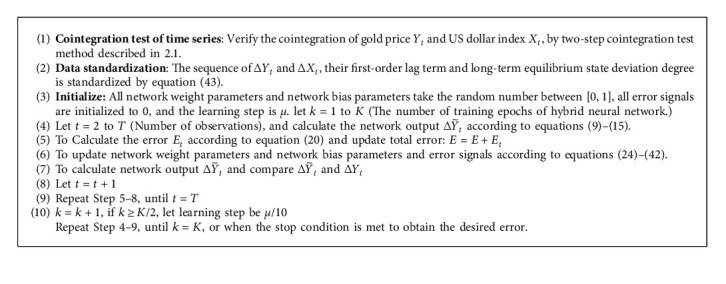
Learning algorithm for nonlinear ECM neural network.

**Table 1:** ADF test results.

Variable	DF statistical quantity	*P* value
Gold price	−0.6388	0.4125
US dollar index	1.3839	0.9569
Gold price first-order difference	−14.0765	<0.01
US dollar index first-order difference	−16.6065	<0.01

**Table 2:** Significance analysis of long-term equilibrium model variables.

Independent variable	Coefficient estimation	*T* statistic	*P* value
Constant term	2557.0240	18.05	<0.01
USDX	−8.1930	−5.35	<0.01

**Table 3:** Neural learning results.

No.	No. of nodes		No. of parameters	Goodness of fit statistical indicators				Chi-square test *P* value compared with simplified model
	First hidden layers	Second hidden layers		Loss	MSE	*R* ^2^	log *L*	
1	0	0	5	1.85	0.0156	0.2068	157.68	
2	1	1	12	1.72	0.0145	0.2558	166.33	0.02 (compare with no. 1)
3	1	3	18	1.72	0.0145	0.2556	166.29	
4	1	5	24	1.72	0.0145	0.2561	166.38	1.00 (compare with no. 2)
5	3	1	18	1.72	0.0145	0.2558	166.33	1.00 (compare with no. 2)
6	3	3	28	1.75	0.0147	0.2449	164.41	
7	3	5	38	1.72	0.0145	0.2560	166.36	1.00 (compare with no. 2)
8	5	1	24	1.73	0.0145	0.2541	166.04	
9	5	3	38	1.76	0.0148	0.2422	163.99	
10	5	5	52	1.72	0.0145	0.2559	166.35	1.00 (compare with no. 2)

**Table 4:** Robustness analysis result.

Variables	Year	Best model	
		In perspective of goodness of fit only	Take both goodness of fit and model complexity into consideration
Gold price as *Y*_*t*_ and US dollar index as *X*_*t*_	2021	Nonlinear ECM neural network with 1 and 5 nodes in first and second hidden layers, respectively	Nonlinear ECM neural network with 1 node in first and second hidden layers, respectively
	2020	Nonlinear ECM neural network with 1 node in first and second hidden layers, respectively	Linear ECM model

US dollar index as *Y*_*t*_ and gold price as *X*_*t*_	2021	Nonlinear ECM neural network with 1 node in first and second hidden layers, respectively	Nonlinear ECM neural network with 1 node in first and second hidden layers, respectively
	2020	Nonlinear ECM neural network with 5 and 3 nodes in first and second hidden layers, respectively	Nonlinear ECM neural network with 1 and 3 nodes in first and second hidden layers, respectively

## Data Availability

The experimental data used to support the findings of this study are available from the corresponding author upon request.
